# Critical Role of Sensitized Serum in Rejection of Allogeneic Bone Marrow Cells

**DOI:** 10.4274/tjh.2012.0213

**Published:** 2014-09-05

**Authors:** Lu-Hong Xu, Jian-Pei Fang, Wen-Jun Weng, Hong-Gui Xu

**Affiliations:** 1 Sun Yat-sen University, Sun Yat-sen Memorial Hospital, Department of Pediatrics, Guangzhou, China; 2 Key Laboratory of Malignant Tumor Gene Regulation and Target, Guangzhou, China

**Keywords:** Sensitized serum, Bone marrow cells, Rejection, Transplantation

## Abstract

**Objective:** Humoral immunity has been clearly implicated in solid organ transplantation, but little is known about the relationship between humoral immunity and hematopoietic stem cell transplantation. This study was designed to investigate that relationship.

**Materials and Methods:** Sensitized serum was obtained from a sensitized murine model established by allogeneic splenocyte transfusion. Sensitized serum was incubated with allogeneic bone marrow cells (BMCs) in vitro and the cytotoxicity was evaluated by the complement-dependent cytotoxicity method. Mice were transplanted with allogeneic BMCs incubated with sensitized serum after lethal irradiation. The engraftment was assayed by hematopoietic recovery and chimera analysis. Moreover, mice received passive transfer of sensitized serum 1 day prior to transplantation. Mortality was scored daily after bone marrow transplantation.

**Results:** The in vitro experiments showed that sensitized serum was capable of impairing allogeneic BMCs through the complement-dependent cytotoxicity pathway. The animal studies showed that BMCs incubated with sensitized serum failed to rescue mice from lethal irradiation. The engraftment assay showed that the allogeneic BMCs incubated with sensitized serum were rejected with time in the recipients. Furthermore, the mice died of marrow graft rejection by transfer of sensitized serum prior to transplantation.

**Conclusion:** Taken together, our results indicated that sensitized serum played a critical role in graft rejection during hematopoietic stem cell transplantation.

## OZET

**Amaç:** Solid organ transplantasyonlarında humoral immunitenin önemi ile ilgili bilgilerimiz daha açık olmakla birlikte, humoral immunite ve hematopoetik kök hücre nakli arasındaki ilişki konusunda bilgilerimiz sınırlıdır. Bu çalışmada bu ilişkinin araştırılması hedeflenmiştir.

**Gereç ve Yöntemler:** Sensitize bir fare modelinden allojeneik splenosit transfüzyonu elde edildi. Sensitize serum, allojeneik kemik iliği hücreleri ile in vitro olarak inkübe edildi ve sitotoksisite komplemana bağımlı sitotoksisite metodu ile değerlendirildi. İrradiasyon sonrası farelere sensitize serumda inkübe edilmiş allojeneik kemik iliği hücreleri transplante edildi. Engraftman, hematopoetik düzelme ve kimerizm analizleri ile değerlendirildi. Ayrıca, farelere transplanttan 1 gün önce sensitize serum pasif olarak uygulandı. Kemik iliği transplantasyonu sonrası mortalite günlük olarak skorlandı. 

**Bulgular:** İn vitro çalışmalar göstermiştir ki, sensitize serum komplemana bağımlı sitotoksisite üzerinden allojeneik kemik iliği hücrelerini etkilemektedir. Sensitize serum ile inkübe edilmiş kemik iliği hücreleri fareleri letal dozda irradiasyondan korumakta başarısız olmuştur. Sensitize serum ile inkübe edilen kemik iliği hücreleri alıcılarda zaman içinde rejeke edilmişlerdir. Ayrıca, transplantasyondan önce sensitize serum verilen fareler graft rejeksiyonu nedeniyle ölmüşlerdir.

**Sonuç:** Bu bilgiler ışığında, sensitize serumun hematopoetik kök hücre transplantasyonunda oluşabilecek graft rejeksiyonunda kritik bir role sahip olduğu söylenebilir. 

## INTRODUCTION

Humoral immunity has been identified to play an important role in solid organ transplantation, especially for sensitized recipients. Allograft rejection is correlated with high levels of donor-reactive antibodies in sensitized serum [1,2,3]. However, little is known about the relationship between humoral immunity and hematopoietic stem cell transplantation. Many hematological diseases, such as thalassemia major, sickle cell disease, and aplastic anemia, require long-term transfusion support. Given that these patients receive repeated transfusions from human leukocyte antigen-mismatched donors, they would be sensitized easily and produce high levels of donor-reactive antibodies [4,5,6]. Currently, allogeneic hematopoietic stem cell transplantation is the only available curative treatment for these diseases. Notably, clinical findings showed that these sensitized patients are at high risk of allogeneic donor graft rejection [7,8]. We assume that allogeneic donor cells present in the blood have direct contact with recipient immune cells in the circulation and may activate donor-reactive antibody production. Thus, humoral immunity may play a critical role in marrow graft rejection.

The level of sensitization associated with transfusions is due to white blood cells present in allogeneic blood products [9,10]. Here, a murine model of sensitization was established by repeated transfusions of allogeneic spleen cells. The serum obtained from such a sensitized model contains high levels of donor-reactive antibodies [11]. In the present study, we tried to explore the effect of sensitized serum on engraftment of allogeneic bone marrow cells (BMCs) in vivo. Moreover, the role of sensitized serum in marrow graft rejection was evaluated by passive transfer prior to transplantation. Informed consent was obtained.

## MATERIALS AND METHODS

**Animals**

Male C57BL/6 and BALB/c mice, aged 6 to 8 weeks and weighing 18 to 20 g, were purchased from the Experimental Animal Center of Sun Yat-sen University (Guangzhou, China). All animals were handled and housed in accordance with the guidelines of the Sun Yat-sen University Animal Care and Use Committee.

**Sensitized Serum and Allogeneic BMCs**

Sensitized serum was collected from sensitized mice. Briefly, BALB/c mice were sensitized by repeated transfusions of allogeneic spleen cells from C57BL/6 mice [11,12]. A total of 1x10^6^ nucleated splenocytes (0.1 mL) were transfused to BALB/c mice via their tail vein weekly for 2 weeks (on day -14 and day -7, respectively). The serum was obtained from the sensitized mice on day 0. Non-sensitized serum was obtained from naive BALB/c mice. All sera were heated at 56 °C for 30 min and were frozen at -20 °C for future use. Meanwhile, allogeneic BMCs were collected from femurs and tibias of C57BL/6 mice. The BMCs were cultured in RPMI-1640 medium.

**Complement-Dependent Cytotoxicity Experiment**

The presence of appropriate alloreactive antibodies in sensitized serum was tested by the complement-dependent cytotoxicity (CDC) method. Ten microliters of serum and 10 µL of allogeneic BMCs were added in 96-well culture plates. Wells were washed once after incubation at 37 °C for 30 min. Ten microliters of rabbit complement (One Lambda Company Limited, Canoga Park, CA, USA) was added and incubated for another 30 min. Fluorescent dye ethidium bromide/acridine orange (One Lambda Company Limited) was added and the percent of non-viable cells was determined by manual counting using a fluorescence microscope [13,14].

**Transplantation of BMCs Incubated with Serum **

Allogeneic BMCs were incubated with a 1:10 dilution of sensitized serum or non-sensitized serum for 1 h on ice. The cells were washed and were then labeled with FITC-conjugated goat anti-mouse antibody (BD PharMingen, San Diego, CA, USA). The percentage of binding rate was detected by a flow cytometer (Becton Dickinson, San Jose, CA, USA). Naive BALB/c mice underwent total body irradiation with 800 cGy using cobalt-60 gamma rays before transplantation. After incubation, allogeneic BMCs were washed 3 times prior to infusion of 1x10^7^ BMCs into irradiated BALB/c mice. Naive BALB/c mice only receiving irradiation alone were used as controls. The recipients were maintained on acidified water with added antibiotics (cidomycin, 32x10^4^ U/L and erythromycin, 250 mg/L).

**Engraftment Assay**

Mortality was scored daily. The survival rate was determined 28 days after transplantation. Twenty microliters of peripheral blood collected in 1-mL tubes with buffer was obtained from the tails of recipients every week after transplantation. Cell numbers were measured by a System KX-21 hematology series cell counter (Sysmex Company, Kobe, Japan). BMCs obtained from femurs of the recipients were counted every week after transplantation. For chimera analysis, BMCs were suspended in RPMI 1640 medium and FITC-labeled H-2Db (BD PharMingen) was added to the cells. The cells were set aside for flow cytometric analysis [15].

**Passive Transfer of Serum Prior to Transplantation**

One day before bone marrow transplantation, 100 µL of sensitized serum or non-sensitized serum was injected intravenously into naive BALB/c mice. The mice underwent total body irradiation with 800 cGy using cobalt-60 gamma rays. A total of 1x10^7^ allogeneic BMCs were transfused intravenously into the irradiated mice. Mortality was scored daily after bone marrow transplantation. 

**Statistical Analysis**

Results are expressed as mean ± standard error of the mean, and the data were analyzed with SPSS 16.0. Comparisons between experimental results were made using one-way ANOVA test analysis. Values of log-rank p were determined using the Kaplan-Meier method comparing survival curves and p<0.05 was considered statistically significant.

## RESULTS

**Sensitized Serum was Capable of Impairing Allogeneic BMCs**

The effect of sensitized serum on allogeneic BMCs was evaluated by the CDC method, which represents the intensity of the complement killing reaction. As shown in Table 1, following 30 min of incubation with rabbit complement, sensitized serum in Group D had significantly increased in the intensity of reaction (% dead cells >40%). In contrast, the reaction remained negative for non-sensitized serum in Group C and samples containing no serum in Group B (% dead cells <10%). As expected, without addition of rabbit complement, there was no increased intensity of reaction in non-sensitized serum (Group E) or in sensitized serum (Group A).

**BMCs Incubated with Sensitized Serum Failed to Engraft in Recipients**

BMCs were incubated with a 1:10 dilution of sensitized serum or non-sensitized serum in vitro. The cells were washed and were then labeled with FITC-conjugated goat anti-mouse antibody. By flow cytometric analysis, the percentage of binding rate in the sensitized serum group and non-sensitized serum group was 95.12±2.32% and 4.15±2.17%, respectively, and the differences were statistically significant (p<0.001).

**Figure 1:**
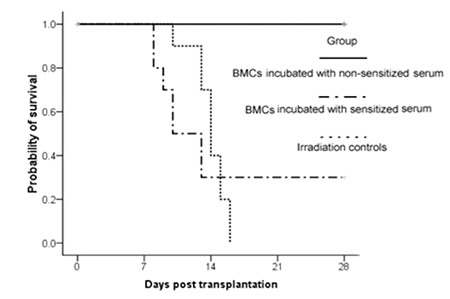
Survival analysis of recipients after irradiation. The irradiation control group was treated with lethal irradiation of 800 cGy, but without bone marrow transplantation. The other irradiated mice received allogeneic bone marrow cells incubated with sensitized or non-sensitized serum, respectively. Each group contained 10 mice. The survival events were monitored daily.

The results of animal survival duration are shown in Figure 1. Each group contained 10 mice. Without transplantation, the mice died at 10 to 16 days after irradiation, with a median of 14 days. The irradiated mice that received allogeneic BMCs incubated with non-sensitized serum remained alive at 28 days. However, 70% (7/10) of the mice receiving allogeneic BMCs incubated with sensitized serum died at 9 to 13 days after irradiation, with a median of 10 days. By log-rank analysis, there was a significant difference between the non-sensitized serum group and the sensitized serum group (p<0.0001), while there was no significant difference between the irradiated control group and the sensitized serum group (p>0.05).

Engraftment of allogeneic BMCs in recipients was assayed at days 7 and 14 after transplantation. The results of hematopoietic recovery and chimera analysis are shown in Tables 2 and 3. The white blood cells, hemoglobin, and platelets in peripheral blood increased over time in mice that received BMCs incubated with non-sensitized serum. In contrast, hematopoietic recovery was noted to be decreased with time in those that received BMCs incubated with sensitized serum. Correlating to peripheral hematopoietic recovery, the number of BMCs per femur and the percentage of H-2Db+ cells in bone marrow increased with time in the non-sensitized serum group but decreased with time in the sensitized serum group.

**Transfer of Sensitized Serum Induced Transplantation Failure**

**Figure 2:**
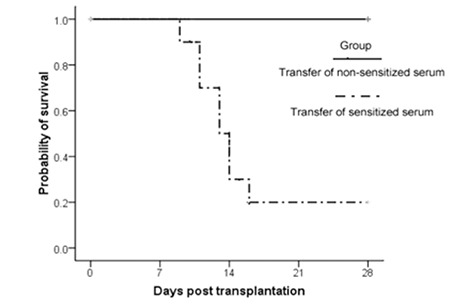
Survival analysis of recipients after bone marrow transplantation. The naive BALB/c mice were transferred with 100 µL of sensitized or non-sensitized serum 1 day before transplantation. All the mice received allogeneic bone marrow cells after lethal irradiation. Each group contained 10 recipients. The survival events were monitored daily.

The irradiated mice were transferred with sensitized serum or non-sensitized serum prior to transplantation. The survival data are recorded in Figure 2. Each group contained 10 mice. All the mice that received non-sensitized serum remained alive at day 28 after bone marrow transplantation. However, 80% (8/10) of the mice that received sensitized serum died at 9 to 15 days after bone marrow transplantation, with a median of 13 days. By log-rank analysis, there was a significant difference between the recipients transferred with non-sensitized serum and those transferred with sensitized serum (p<0.0001). Moreover, the white blood cells, hemoglobin, and platelets in the peripheral blood of dying mice were (0.28±0.08)x109/L, 80.4±8.02 g/L, and (38.8±7.25)x109/L, respectively. These values indicated that the mice died from marrow graft rejection. 

## DISCUSSION

CDC is a reaction in which an antibody bound to its antigen activates a reaction cascade of the complement system. Our results showed that the cytotoxic index of the CDC experiment in Group D was significantly higher than that in Group C (p<0.0001), indicating that sensitized serum was capable of impairing allogeneic BMCs through the CDC pathway. Moreover, the cytotoxic index in Group E and Group A was negative, indicating that the reaction depended on complement. In the binding experiment, our results suggested that the donor-reactive antibodies in sensitized serum were binding 95% of BMCs with 1:10 dilution. BMCs incubated with sensitized serum could not rescue mice from lethal irradiation. The engraftment assay demonstrated that allogeneic BMCs incubated with sensitized serum were rejected along with time in recipients. Furthermore, 80% (8/10) of the mice died of marrow graft rejection by transfer of sensitized serum prior to transplantation, indicating that sensitization could be transferred by donor-reactive antibodies in serum. All of the results indicated that sensitized serum played a critical role in graft rejection during hematopoietic stem cell transplantation.

The mechanism of donor graft rejection by sensitized serum remains unclear. It has been suggested that donor-reactive antibodies in sensitized serum may induce primary endothelium injury through complement-dependent pathway and complement-independent pathway antibody-dependent cell cytotoxicity in solid organ transplantation. Endothelium injury results in progressive tissue injury and final graft function loss [16,17,18]. Otherwise, the hematopoietic stem cells present in circulation are prone to being impaired by donor-reactive antibodies. Several studies have found that humoral immunity plays a critical role in the rejection of allogeneic marrow in sensitized recipients. Preformed antibody is the initial and major barrier to bone marrow engraftment in allosensitized recipients. The rejection might be dependent upon host FcR+ cells [19,20]. Moreover, alloreactive memory T cells are also considered to contribute to rejection of donor BMCs in sensitized recipients [21]. Recently, donor-reactive antibodies in sensitized serum were found to be associated with graft failure in patients with hematopoietic stem cell transplantation [22]. Our previous data showed that high levels of donor-reactive antibodies were found in serum of patients with beta-thalassemia major. In addition, the sensitized serum had an inhibitory effect on proliferation and differentiation of hematopoietic stem cells in vitro [23].

It is urged to develop new strategies to prevent and reduce the risk of graft rejection mediated by humoral immunity. The managements include inhibition and depletion of antibody-producing cells, removal or blockage of preexisting or newly developed antibodies, and impediment or postponement of antibody-mediated primary and secondary tissue injury [16,24,25]. We suggest that leukocyte-depleted blood components be used for patients requiring long-term blood transfusions. Donor-reactive antibodies should be routinely monitored for sensitized patients in allogeneic transplantation. Intervention therapies such as removal or blockade of serum antibodies may be considered before transplantation [26]. In clinical settings, 4 sensitized patients were reported to be treated with a combination of rituximab and plasma exchange. This intervention decreased the antibody levels substantially in 2 patients, who both achieved marrow engraftment [27]. Further investigations are required to develop new strategies for sensitized transplantation.

**Conflict of Interest Statement**

The authors of this paper have no conflicts of interest, including specific financial interests, relationships, and/ or affiliations relevant to the subject matter or materials included.

**Acknowledgments:** This work was supported by the National Natural Science Foundation of China (81100370).

## Figures and Tables

**Table 1:**
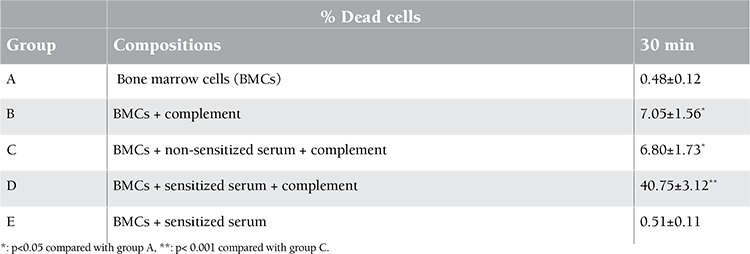
Intensity of reaction in complement-dependent cytotoxicity experiment (n=5).

**Table 2:**
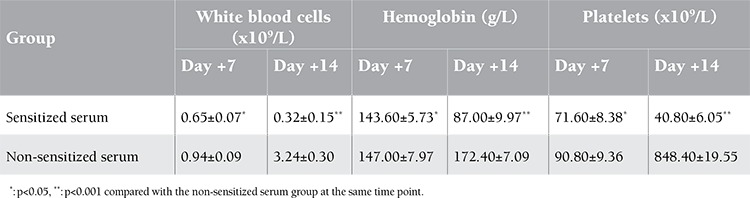
Hematopoietic recovery of recipients post transplantation (n=5).
